# Chip‐Level Voltage Controlled Magnetic p‐Bits Array with Variation Compensation for Stochastic Neural Network Computation

**DOI:** 10.1002/advs.78604

**Published:** 2026-11-10

**Authors:** Jiachen Bao, Songsong Li, Ruizhi Ren, Zheng Zhu, Di Wu, Jiayong Wang, Minggao Zuo, Mingke Li, Duanzihua Cheng, Jianyu Lv, Lintong Zou, Chuyue Liu, Wangyang Hu, Yi Cao, Yuesheng Li, Guanghua Yu

**Affiliations:** ^1^ School of Materials Science and Engineering University of Science and Technology Beijing Beijing China; ^2^ College of Sciences Northeastern University Shenyang China; ^3^ Institute of Integrated Circuits Shanghai University Shanghai China; ^4^ Key Laboratory of Advanced Materials and Devices for Post‐Moore Chips Ministry of Education University of Science and Technology Beijing Beijing China

**Keywords:** algorithm, artificial intelligence, boltzmann machine, chip, electronic engineering, electronic spin, magnetism, materials science, tunnel junction

## Abstract

The increasing computational and energy‐efficiency demands of artificial intelligence are driving the development of new computing paradigms based on emerging memory devices. Probabilistic bits (p‐bits) based on magnetic tunnel junctions (MTJs), benefiting from energy efficiency, CMOS compatibility, and fast sampling, show broad potential in stochastic neural networks. However, unavoidable device‐to‐device variations in MTJ switching probabilities hinder the realization of scalable p‐bit chips. Here, we integrate a high‐density array of voltage‐controlled magnetic anisotropy MTJs (VCMA‐MTJs) and employ a tail truncation strategy to compensate for their variations in probabilistic switching ranges, enabling chip‐level p‐bit functionality. Our VCMA‐MTJ chip (VM‐chip) successfully implements restricted Boltzmann machines (RBM) and deep belief networks (DBN) for image recognition, anomaly detection, image completion, and image generation, demonstrating the practical utility of this chip in stochastic neural networks. Furthermore, we propose a dual‐control strategy that tunes both the amplitude and duration of the voltage pulse, significantly enhancing network performance and performing comparably to conventional software‐generated pseudo‐random numbers. Our work enhances the tolerance of probabilistic computing to variations in MTJ switching probabilities, offering flexibility in device selection and array integration. This approach may provide a promising pathway toward scalable p‐bit hardware and cost‐effective manufacturing in future probabilistic computing systems.

## Graphical abstract

A chip‐level VCMA‐MTJ array is demonstrated for stochastic neural‐network computing. Combined with a tail‐truncation strategy to compensate for device‐to‐device variations, it establishes a hardware–software co‐design approach for scalable probabilistic computing.

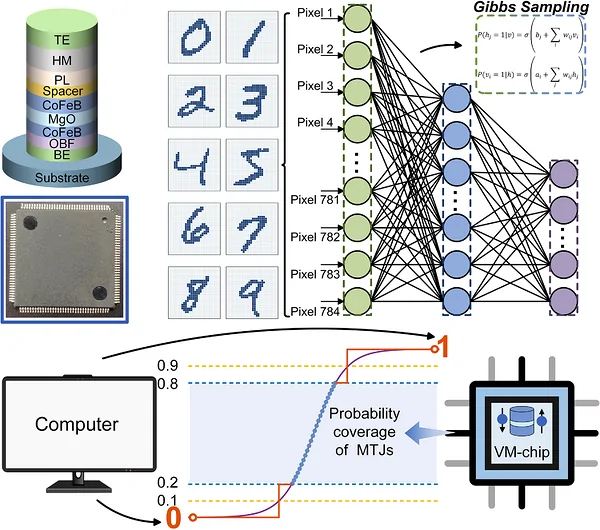

## 1 Introduction

In recent years, the rapid development of artificial intelligence has driven a continuous expansion in the scale of foundational models, particularly those based on stochastic neural networks [1, 2, 3, 4, 5, 6]. In stochastic neural network computation, in addition to large‐scale matrix operations, random sampling constitutes an indispensable core component [7, 8]. This process is typically realized using probability bits (p‐bits), which are binary stochastic neurons capable of outputting 0 or 1 with a given probability, thereby enabling the randomized operations required for probabilistic inference [9, 10]. Currently, mainstream p‐bits are primarily based on CMOS technology and are realized through combinations of a large number of transistors or programmable logic arrays (FPGAs) [11, 12, 13, 14]. However, as network scales increase, these approaches face growing limitations in terms of device count and power consumption [15, 16, 17]. Meanwhile, the separation between p‐bit generation and computation in conventional digital architectures further exacerbates the efficiency bottleneck in large‐scale probabilistic algorithms [18, 19, 20]. To overcome these limitations, researchers have been exploring device‐level stochastic computing approaches based on emerging memory devices, where p‐bit generation is directly implemented in physical devices and can be naturally integrated with computation functions [21, 22, 23, 24].

Among the various candidate solutions, magnetic tunnel junction (MTJ) is naturally suited as the physical carrier of probability bits because the magnetization direction of their free layer can represent binary 0 or 1 [25]. Besides, MTJs offer CMOS compatibility, high‐speed sampling, and low power consumption, making them an ideal hardware platform for implementing p‐bit [26, 27, 28]. By leveraging spin‐transfer torque (STT) modulation, MTJs can achieve a tunable probability range from 0% to 100%, enabling them to serve as physical nodes in random neural networks or Ising models to perform Markov chain Monte Carlo (MCMC) sampling [29, 30]. This capability allows efficient hardware‐level solutions to a variety of combinatorial optimization and probabilistic inference problems, such as integer factorization and the traveling salesman problem (TSP) [10, 31, 32, 33, 34, 35]. Compared with STT, in which the write current flows directly through the MTJ and degrades device lifetime, spin–orbit torque MTJs (SOT‐MTJs) employ a current‐path separation scheme that significantly improves device endurance, thereby making them well suited for probabilistic applications such as invertible logic and restricted Boltzmann machine (RBM) [34, 36, 37, 38, 39, 40, 41, 42]. Nevertheless, reliable switching in STT‐MTJ and SOT‐MTJ requires large write currents, which induce pronounced Joule heating and increased energy consumption, which consequently limits their deployment in large‐scale, energy‐efficient applications [43, 44]. Voltage‐controlled magnetic anisotropy MTJs (VCMA‐MTJs) address this issue by modulating the magnetic anisotropy energy barrier via an electric field, enabling magnetization control with only a minimal current, leading to lower switching energy consumption [45, 46, 47]. However, device‐to‐device variations induced by fabrication imperfections, temperature fluctuations, and write‐voltage instabilities significantly limit the functional realization of VCMA‐MTJs in large‐scale arrays. Physically, these variations manifest as differences in stray fields and non‐uniform magnetization precessional dynamics, leading to deviations in the switching probability distribution across devices. To bypass the detrimental interference of such process non‐uniformity on precise probability control, previous works typically employed multiple exclusive‐OR (XOR) logic layers or alternative mathematical treatments for post‐processing correction [48, 49]. Nevertheless, this approach is applicable only to random number generators with a fixed 50% switching probability, rendering it fundamentally incapable of constructing a single‐device p‐bit with continuously tunable probabilities. Currently, such large‐scale, chip‐level p‐bits array with continuously tunable probabilities spanning from 0% to 100% is still lacking, which severely constrains the scalability of probabilistic computing systems.

In this work, we constructed a chip‐level p‐bit array using VCMA‐MTJ and employed a tail truncation method to compensate for device‐to‐device variations in probability ranges. Based on this platform, we successfully implemented RBM and DBN models, accomplishing tasks including image recognition, anomaly detection, image completion, and image generation. The tail truncation improved the accuracy of image recognition by eightfold. Furthermore, we developed a dual‐control strategy based on simultaneous tuning of the voltage pulse amplitude and duration, thereby enhancing the performance of the network and achieving performance comparable to that of conventional software‐based pseudorandom sampling, which demonstrates the practicality and application potential of the VCMA‐MTJ chip (VM‐chip). Compared with CMOS circuits, the VM‐chip requires only one‐tenth of the energy per generated bit, achieves an exponential reduction in transistor resource usage, and provides true randomness. We anticipate that the proposed approach may improve process tolerance and offer greater flexibility in device selection and array integration, potentially facilitating the development of scalable probabilistic computing chips and more cost‐effective manufacturing in the future.

## 2 Results and Discussion

### 2.1 Magnetic Properties and Probability Range Variations of VCMA‐MTJs

The multilayer structure of the VCMA‐MTJ is illustrated in Figure 1a, which consists of a conventional CoFeB/MgO/CoFeB trilayer tunneling junction, forming perpendicular magnetic anisotropy (PMA) at the interfaces. In addition, a relatively thick MgO barrier effectively suppresses unwanted contributions from STT currents and enables low‐energy voltage‐driven switching of the free layer magnetization [50]. Figure 1b shows the TMR response of VCMA‐MTJ under a perpendicular magnetic‐field sweep, where the free layer reversibly switches between the parallel (P) and antiparallel (AP) states as the external field varies. The VCMA‐MTJ demonstrates a high‐quality magnetoresistance window, with a maximum TMR of 177.21% and a TMR of 168.43% at zero external field, corresponding respectively to switching ratios (R_AP_/R_P_) of 277.21% and 268.43%, highlighting the excellent barrier quality of MgO that is sufficient to meet the resistance contrast and read/write reliability requirements of probabilistic computing. Figure 1c illustrates the VCMA effect used to induce magnetization switching in the MTJ. At equilibrium (zero applied voltage), the energy barrier separates the two stable magnetization states of the free layer (P and AP). Upon the application of a voltage pulse, the energy barrier is reduced, causing the free‐layer magnetization to precess around an in‐plane axis. By controlling the duration of the applied voltage, the magnetization ultimately settles into different stable states, enabling probabilistic switching [51]. To further investigate the switching probability difference between the P and AP magnetization states, the VCMA‐MTJ switching probability as a function of external magnetic field under different voltage biases was measured, as shown in Figure 1d. Under equivalent voltage conditions, the AP‐to‐P and P‐to‐AP probability curves exhibit a clear mirror symmetry, indicating that the device possesses a highly symmetric energy barrier between the two states. Consequently, regardless of the initial state being P or AP, the same voltage pulse can achieve switching with equal probability, eliminating the need for additional initialization and reducing the complexity of probability calculations. Figure 1e shows the probability density distributions of a large number of VCMA‐MTJs in the P and AP states, providing an assessment of the operational reliability of the VM chip. Both the P and AP states exhibit well‐defined Gaussian distributions with no overlap between the two resistance states. The mean resistances of the R_P_ (R_AP_) is 93.41 kΩ (259.06 kΩ) with corresponding standard deviations of 6.42 kΩ (14.14 kΩ). From these values, the discrimination metric ($D=\frac{\left|\mu R_{P}-\mu RA_{P}\right|}{\sigma_{P}+\sigma_{AP}}$) is determined to be 8.06, and the center‐to‐center distance between R_P_ and R_AP_ exceeds 25 times the standard deviation of the low‐resistance state. The wide read margin and high D value exceed the minimum reliability threshold for probabilistic operations, resulting in an extremely low bit‐error rate during device readout.

**FIGURE 1:**
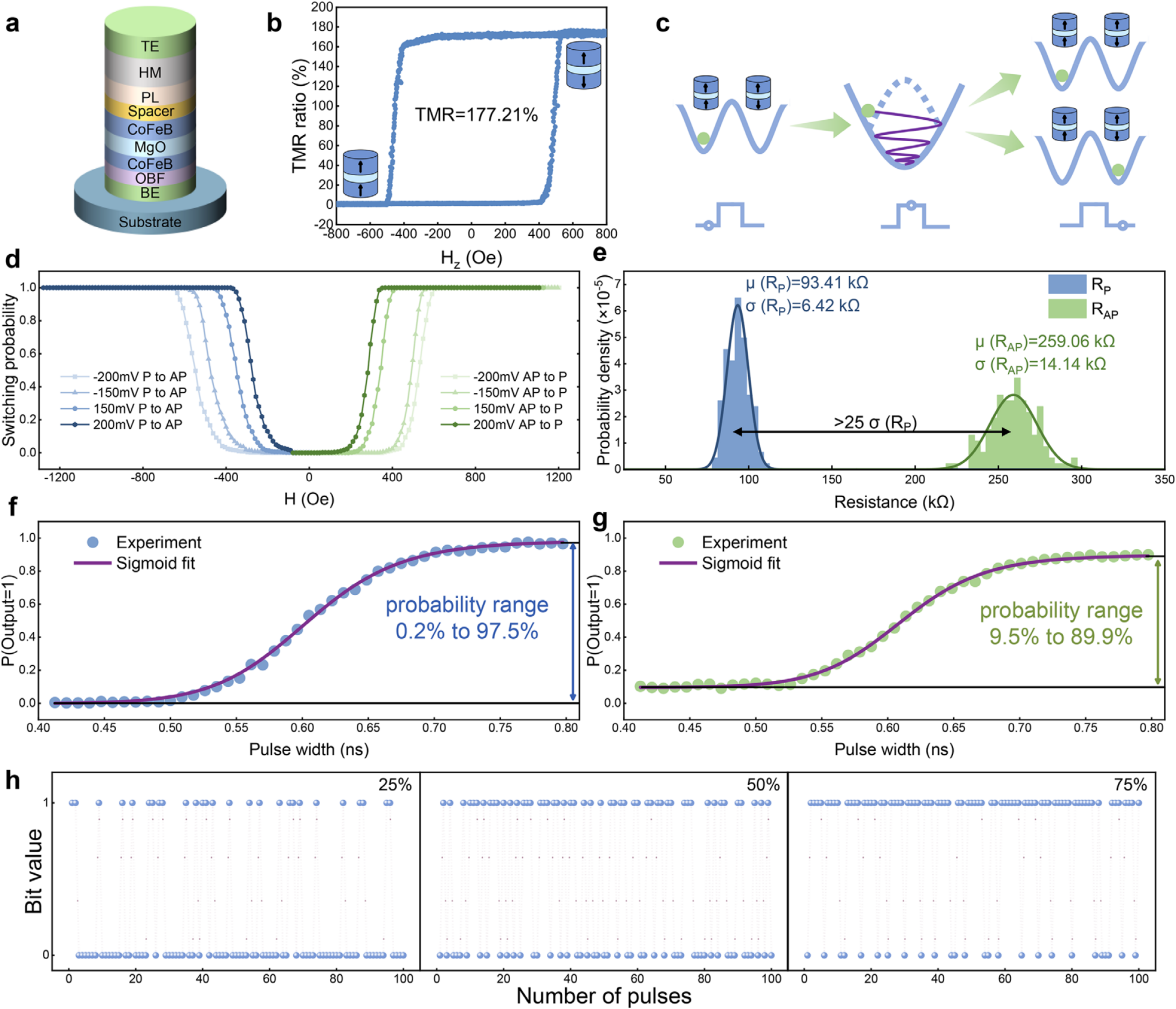
Magnetic Characteristics and Probabilistic Switching of VCMA‐MTJ Devices. a, Multilayer stack structure of the VCMA‐MTJ. b, TMR characteristic curve of VCMA‐MTJ. The device exhibits a TMR ratio of 177.21%, which corresponds to an on/off ratio of 277.21%. c, Schematic of the VCMA‐MTJ switching mechanism. d, Switching probability versus magnetic field under various bias voltages. e, Device‐to‐device (D2D) distribution of the parallel (blue) and antiparallel (green) resistance states. The separation between the two states exceeds 25σ(R_P_). f and g: switching probability curve and the corresponding sigmoid fit of the wide and narrow probability range VCMA‐MTJ at a write voltage of 1.9 V. h, Sequence of 100 bits generated at probabilities of 25%, 50%, and 75%.

A switching of VCMA‐MTJ from R_AP_ to R_P_ or from R_P_ to R_AP_ under a single applied voltage pulse is recorded as an output signal of 1, whereas maintaining the original resistance state is recorded as 0, thereby enabling random sampling. As shown in Figure 1f, the probability curve of the device was measured and fitted with a sigmoid function for use as the activation function in stochastic neural networks, demonstrating an almost full probability range (0.2% to 97.5%). The relationship between probability and pulse width can be expressed as:

$$P=\frac{1}{1+e^{-\beta \left(W_{v}-W_{0}\right)}}$$

It is worth noting that not all MTJs exhibit a probability range approaching 0% to 100%, with the device shown in Figure 1g presenting a noticeably narrower tunable probability window. The observed monotonic sigmoid‐like characteristics between 2 MTJs are attributed to the narrow window of applied pulse widths (less than 1 ns), which primarily captures the first half‐period of this precessional oscillation. Consequently, the distinct profiles between wide‐range and narrow‐range MTJs fundamentally originate from the device‐to‐device variations in their precessional dynamics driven by process‐induced non‐uniformities. Probability switching curves of more functional devices and cycle‐to‐cycle variations of MTJ‐1 can be found in Figures S1 and S2, respectively. Under a write voltage of 1.9 V, sequences of 100 random bits were generated at pulse widths of 0.56, 0.60, and 0.64 ns (corresponding to p = 25%, 50%, 75%), as shown in Figure 1h. For each pulse condition, the resulting bitstreams show no evident bias drift or clustering behavior, demonstrating good distribution uniformity and temporal stability. In addition, the autocorrelation and cross‐correlation of the generated bits at a switching probability of 0.5 were characterized, as shown in Figure S3. The generated bitstream was also evaluated using the NIST SP 800–22 statistical test suite, with the detailed results provided in Table S1. It is demonstrated that the VM‐chip can stably generate random bits with well‐defined statistical properties, thereby providing a reliable device foundation for probabilistic computing and sampling.

### 2.2 VM‐Chip Testing System

Figure 2 illustrates the overall architecture of the VM‐chip testing system, and the photograph of the experimental prototype is provided in Figure S4. The testing system consists of a computer, an MCU, and the VM‐chip, which together form the complete measurement platform. As shown in Figure 2a, the host computer communicates with the MCU through a serial interface to transmit control commands and receive response data. Figure 2b presents the primary functional modules of the MCU, which receives control commands from the host computer and sequentially performs cell addressing, pulse control, and resistance‐state readout after the pulse operation. It should be noted that the MCU does not directly generate nanosecond‐scale write pulses but instead supplies the VM‐chip with pulse‐control parameters, including pulse width and amplitude, through a digital configuration. Based on the configuration provided by the MCU, the on‐chip pulse‐generation module activates weighted resistive branches that modify the effective discharge resistance, which ultimately determines the pulse duration. Figure 2c shows a photograph of the fabricated VM‐chip along with the structure of its internal memory array, while the photograph of the unpackaged wafer is provided in Figure S5. The chip incorporates a 32 Kb (256 × 128) 2D VCMA‐MTJ array (cross‐sectional SEM image shown in Figure 2d) that can independently perform write and read operations for random‐bit generation and probabilistic sampling. More SEM images at various magnifications are provided in Figure S6. Figure 2e depicts the simplified structure of a single array cell which contains a VCMA‐MTJ together with its associated access transistor. The adjustable V_write_ modulates the interfacial magnetic anisotropy barrier through the VCMA effect to induce magnetization switching for writing, while the resistance of the MTJ is subsequently measured using V_read_ = 0.6 V and V_load_ = 0.2 V to distinguish between the P and AP magnetization states.

**FIGURE 2:**
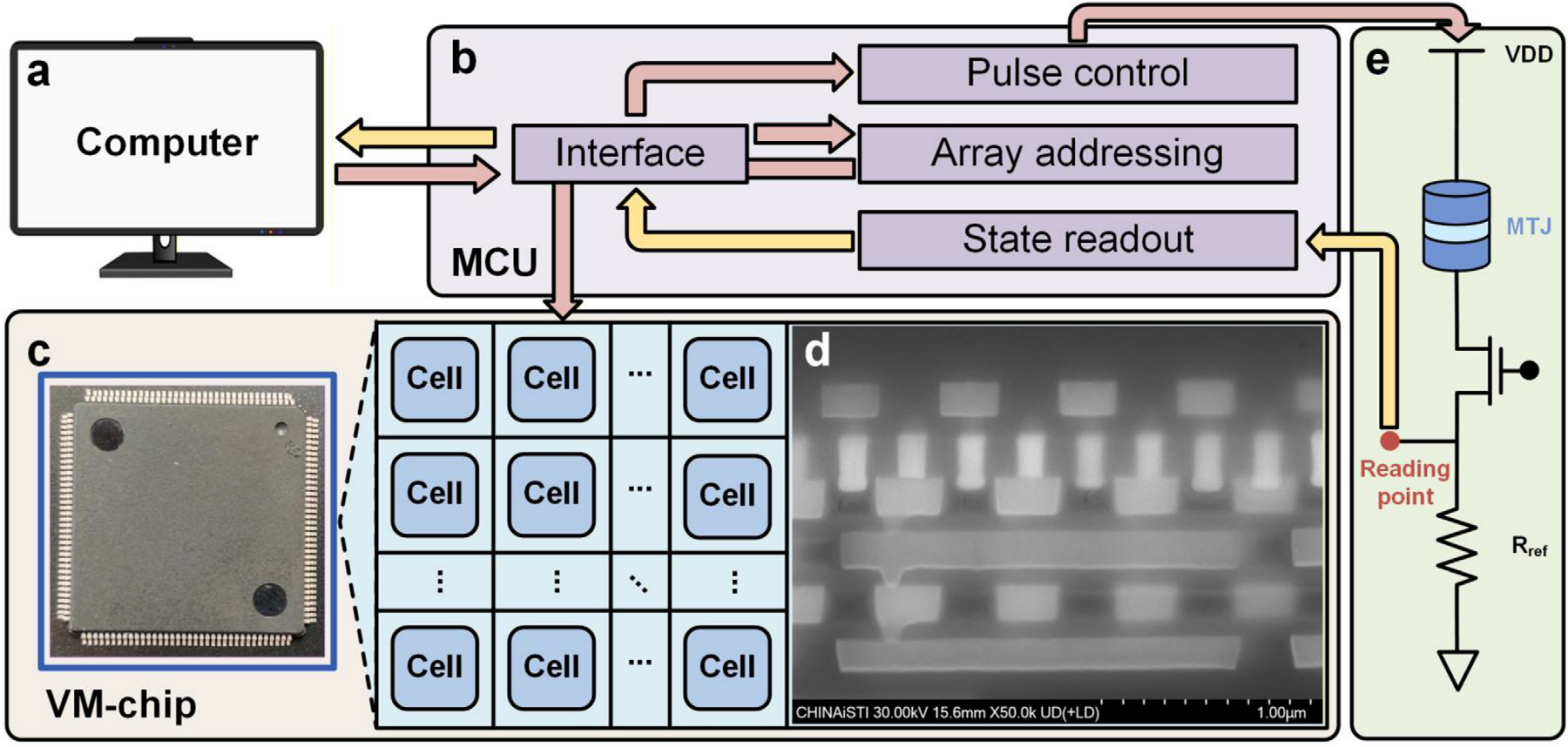
Complete architecture diagram of the VM‐chip testing system. a, The host controls the MCU to perform VCMA‐MTJ device addressing, voltage state readout, and pulse parameter configuration. b, Layout of the VM‐chip, where the yellow dot arrays represent the VCMA‐MTJ devices. c, Real photograph of the VM‐chip, which consists of multiple cells integrating MTJ devices. d, Cross‐sectional SEM image of the fabricated VCMA‐MTJ array. e, Circuit diagram of the MTJ‐containing cell. The magnetization switching is induced by modulating the magnetic anisotropy via the duration of the adjusted V_write_. V_read_, V_load,_ and R_ref_ are set to 0.6, 0.2 V, and 100 kΩ, respectively.

### 2.3 Impact of Probability Range and Compensation Strategies on Image Recognition

RBMs are widely employed for pretraining on unlabeled data due to their strong performance in unsupervised feature learning [14, 52, 53]. During the training phase, in addition to deterministic operations such as matrix multiplications, RBMs rely on a Gibbs sampling process based on conditional probability distributions defined by the model parameters to stochastically update the binary states of neurons. In this sampling process, the conditional probabilities can be naturally expressed in the form of a sigmoid function, which maps the local input to the probability of a neuron being in the active state. Since the stochastic switching behavior of MTJ devices likewise exhibits a sigmoid‐shaped probability response, the proposed VM‐chip is inherently well suited to meet the large‐scale Gibbs sampling demands of RBM. More details on the RBM and DBN models can be found in Note S1.

Figure 3a illustrates the RBM architecture for MNIST recognition. Each 28 × 28 image is binarized (pixels > 0.5 are set to 1, and 0 otherwise) and flattened into a 784‐dimensional vector to serve as the input to the visible layer of the RBM. The hidden layer includes 256 nodes, which learn latent representations of the input data in unsupervised training through bidirectional weighted connections with the visible layer. The resulting hidden activations are subsequently fed into a supervised classifier to perform categorization over the ten digit classes (More algorithm and details are provided in Note S2 and Figure S7). Notably, the weight computations, gradient calculations, and parameter updates in the RBM are performed by the host computer, whereas only the sampling of the hidden and visible states is carried out on the VM‐chip. The workload distribution and corresponding execution times are summarized in Tables S2 and S3, respectively.

**FIGURE 3:**
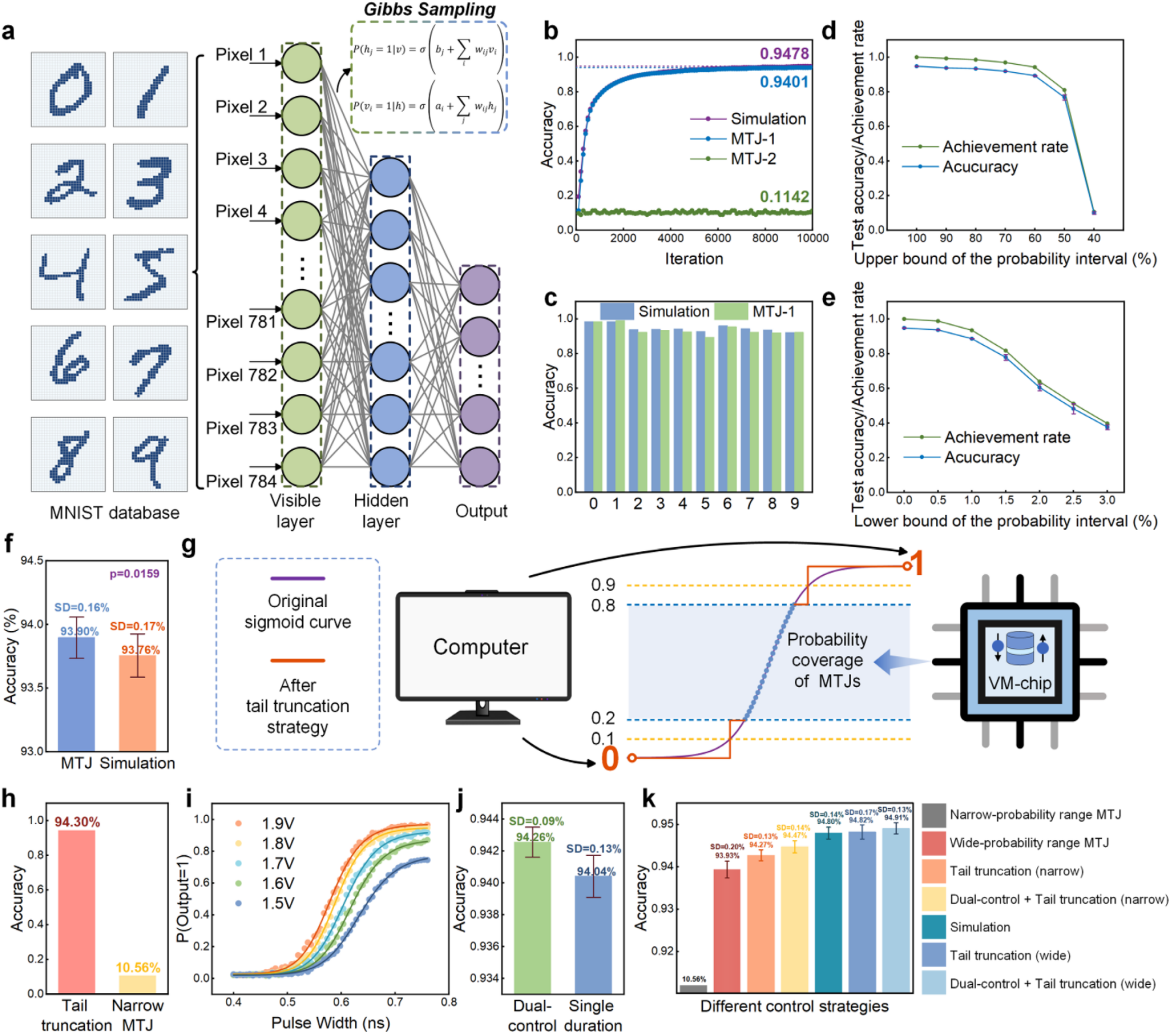
Implementation of Restricted Boltzmann Machine for image recognition based on VM‐Chip. a, Schematic of the RBM architecture. The visible layer and hidden layer contain 784 nodes and 256 nodes, respectively. The weight matrix between the two layers is updated via Gibbs sampling. The output layer consists of 10 nodes representing digits 0 to 9. b, Comparison of accuracy over the training iterations between computer simulation and MTJ devices with different probability ranges. c, Comparison of classification accuracy for each digit between computer simulation and MTJ‐1. d and e, Compute simulations were conducted to examine the dependence of accuracy and the achievement rate on the upper (upper) bound of the probability interval, with the lower (upper) bound fixed at 0 (1). f, Comparison of accuracy between MTJ devices and computer simulation in an image recognition task within the same probability range. Data are presented as mean ± standard deviation, with the p‐value indicating the statistical significance of the difference between the two groups. g, Schematic of the tail truncation strategy. Random bits within the probability range of 0.2 to 0.8 are generated by the VM‐chip. When the desired probability falls within 0.1 to 0.2 (0.8 to 0.9), the VM‐chip output at 0.2 (0.8) is used as a substitute. For probabilities below 0.1 (above 0.9), the software deterministically outputs 0 (1). h, Accuracy comparison of the MTJ with and without the tail truncation strategy. i, Switching probability curves of MTJ under different V_write_ and their corresponding sigmoid fits. j, Comparison of accuracy between the dual‐control co‐control and the single pulse control. Data are presented as mean ± standard deviation. k, Comparison of accuracy under different control strategies. Data are presented as mean ± standard deviation.

Figure 3b presents the RBM training accuracy curves obtained using computer simulation (0% to 100%) and VCMA‐MTJs (MTJ‐1 corresponding to 0.2% to 97.5%, MTJ‐2 corresponding to 9.5% to 89.9%) as random bit inputs. The computer simulation random source, serving as the ideal baseline, reaches a convergence accuracy of 94.78% at 10 000 iterations. MTJ‐1, with a wide probability range, enables effective sampling in both the positive and negative phases, allowing its learning curve to closely match the simulated reference and yielding a final accuracy of 94.01%. Conversely, the constrained probability interval of MTJ‐2 hampers Gibbs sampling during training, resulting in accuracy remaining at approximately 10% with no observable improvement across iterations. Figure 3c provides a comparison of the per‐class recognition accuracy for RBMs trained with the simulated random source and with MTJ‐1. The two configurations exhibit highly consistent performance across all digit categories, each exceeding an accuracy of 90%, which suggests that the stochastic behavior of the physical MTJ‐1 is adequate for enabling stable RBM inference and probabilistic computing. In addition, we implemented MNIST image recognition using a DBN and performed Fashion‐MNIST recognition using an RBM, both based on MTJ‐1 and Simulation, which are provided in Figure S8, further demonstrating the versatility of MTJ‐1 across different network architectures and datasets.

Considering that the width of the probability interval may affect recognition performance, we further conducted simulations under different interval settings. With the lower bound fixed at 0 (or the upper limit fixed at 1), various probability intervals were constructed by adjusting the corresponding upper (or lower) bound, and the results are shown in Figure 3d,e. To quantitatively evaluate the influence of these intervals on recognition accuracy, the concept of achievement rate is introduced, defined as the ratio between the recognition accuracy obtained under a given probability interval and that obtained under the baseline interval (0% to 100%). When the achievement rate exceeds 0.98, the recognition performance is considered not to exhibit noticeable degradation. Interestingly, variations in the upper bound of the probability interval have only a minor impact on overall performance. Even when the interval is restricted to 0% to 80%, the achievement rate remains above 0.98. In contrast, the lower bound is much more sensitive, and when the interval is shifted to 1% to 100%, the achievement rate drops to 0.93. To explain this asymmetry, the activation probability distribution used during the RBM sampling process is analyzed (see Figure S9), which shows that probabilities below 10% account for a substantial portion. This is primarily due to the characteristics of the MNIST dataset, in which most background pixels remain inactive while only a small fraction of pixels contribute to the actual digit shape. As a result, the model frequently operates within low activation probability ranges during inference and reconstruction. Moreover, the features responsible for capturing digit edges and fine details also rely heavily on low activation probabilities, making the recognition performance particularly sensitive to the lower bound of the probability interval. Figure 3f compares the accuracy of the MTJ‐1‐based physical random source with that of the software‐generated random source under the same probability interval of 0.2%‐97.5%. The MTJ‐based approach achieves accuracy comparable to that of the software‐generated source, with a slightly higher mean accuracy across 20 paired training runs (p = 0.0159). More statistical analyses are provided in Table S4. These results indicate that the MTJ can provide a physical stochastic source for image recognition with performance comparable to that of a software‐generated random source.

To address the insufficient coverage of the probability range, we propose a tail truncation strategy, as illustrated in Figure 3g. Within the 20% to 80% probability range (blue region), random sampling is performed using narrow‐range VCMA‐MTJs. For the 0.1 to 0.2 (0.8 to 0.9) range, sampling is conducted at probabilities of 0.2 (0.8). When the probability falls below 0.1 or exceeds 0.9, the software controlled by the host deterministically outputs 0 (1) to ensure that the entire probability range is effectively covered. With the tail truncation strategy, the performance of narrow‐range VCMA‐MTJs in image recognition is significantly enhanced, as depicted in Figure 3h. Compared with using a single device, an approximately 9 times improvement in accuracy is achieved, approaching the level obtained in simulations. To further evaluate the robustness of the proposed approach against device‐to‐device variation, we performed the same task using 28 additional MTJ devices from the chip individually. All 28 devices achieved recognition accuracies above 94%. Furthermore, the 28 devices were jointly used for computation, and the experiment was independently repeated 10 times, with recognition accuracy consistently remaining above 94% (as shown in Figure S10). Beyond Boltzmann machine‐based computer vision tasks, the computational versatility of our VM‐chip was successfully evaluated on typical combinatorial optimization problems, including integer factorization and the Traveling Salesman Problem (TSP); the detailed algorithms are elaborated in Notes S3 and S4. In the integer factorization task, the successful decomposition of the composite number 35 was stably achieved using both MTJ‐1 and MTJ‐2 (as shown in Figure S11), demonstrating the robust architectural resilience of the framework under non‐ideal hardware constraints without requiring strict boundary limitations. In contrast, the TSP solver demonstrates a much higher sensitivity to the non‐idealities of physical devices, particularly the incomplete probability intervals (see Figure S12). These device deficiencies introduce persistent stochastic perturbations that severely hinder late‐stage energy convergence and significantly inflate the iteration counts required to hit the global minimum, an optimization bottleneck that is effectively mitigated by the introduction of our proposed tail truncation strategy. This tail truncation strategy can be viewed as a hardware–software co‐design architecture that aims to relax the stringent requirements for device‐to‐device uniformity. Its underlying motivation lies in the varying sensitivity of stochastic neural networks to sampling probabilities. During Gibbs sampling, the network exhibits a degree of robustness to small deviations in the intermediate probability range (e.g., 0.1–0.9), whereas deterministic handling of tail probabilities approaching zero can help prevent the loss of critical feature states. Based on this characteristic, the strategy uses physical MTJs for stochastic sampling within their reliable probability windows, while offloading the deterministic handling of extreme probabilities to software. However, this strategy also has limitations, as some target probabilities cannot be precisely realized. For example, target probabilities in the range of 0.1–0.2 are approximated using a probability of 0.2, causing the actual sampling probability to deviate from the algorithmically specified value. Owing to the inherent fault tolerance of probabilistic algorithms, no obvious adverse effects on solution quality or convergence speed were observed in the applications investigated in this work. Therefore, this strategy represents a trade‐off between probability realization accuracy and hardware implementation requirements, sacrificing the precise realization of some target probabilities in exchange for reliable access to deterministic boundary values, thereby reducing the requirements for device‐to‐device uniformity.

In the VM‐chip, the pulse duration is controlled by tuning the effective resistance through parallel combinations of multiple resistors (see Note S5 and Figure S13). Although this discretized scheme cannot provide an exact pulse width for every target probability, it already offers sufficient resolution for probabilistic computing. Nevertheless, we still introduce a dual‐control strategy that not only relies on pulse duration‐based modulation but also incorporates voltage amplitude as an additional control dimension to further improve precision, thereby enabling the VM‐chip to support applications with potentially higher accuracy requirements. Figure 3i shows the probability characteristics of the VCMA‐MTJ as a function of pulse width under different V_write_. At a higher voltage (1.9 V), the probability curve becomes steeper and spans a wider probability range (nearly 0% to 100%). In contrast, at a lower voltage (1.5 V), the upper bound of the achievable probability decreases, and the curve becomes flatter, covering a narrower range (approximately 0% to 75%), which indicates that the voltage amplitude effectively compresses the probability response, providing higher resolution within specific probability intervals. This behavior may be attributed to the reduced VCMA‐induced modulation of magnetic anisotropy at lower voltages, which may be insufficient to completely eliminate the energy barrier and thus limit the maximum achievable switching probability [54, 55]. This dual‐control strategy thereby enables improved recognition performance, as shown in Figure 3j. Compared with pulse‐duration‐only control, dual control provides greater flexibility in selecting probability setpoints, thereby improving the performance of probabilistic computing.

Figure 3k compares the performance of various control strategies on the MNIST image recognition task. As shown, relying solely on narrow‐range MTJs yields an accuracy of only 0.11, which is insufficient to support the sampling requirements of the RBM. In contrast, wide‐range MTJs can already fulfill the sampling task, although their performance still falls noticeably short of the ideal simulation baseline. With the introduction of the tail truncation strategy, the accuracy improves for both narrow‐range and wide‐range devices, further approaching the simulation reference and even slightly surpassing it in certain cases. Building upon this, the incorporation of the dual‐control strategy provides additional improvement by enabling higher‐probability resolution within local probability segments. It should be noted that “narrow range” and “wide range” are used here to represent two representative endpoints of device probability response capability. In practice, most VCMA‐MTJs in VM‐chip exhibit probability ranges that lie between these two extremes.

### 2.4 VM‐chip Based Anomaly Detection

Leveraging the feature extraction and classification capabilities of RBMs, we further investigated their application in anomaly detection. Unlike image recognition, anomaly detection not only requires the model to capture the typical patterns of the data but also to sensitively identify samples that deviate from these patterns. To this end, the digit “1” from the MNIST dataset was treated as normal samples, and 2% of samples from each of the other digits were randomly selected as abnormal samples, simulating the real‐world scenario in which anomalous samples are far fewer than normal ones (detailed algorithm in Note S6). Considering that the AUC of the ROC curve (see Figure S14a) may be overly optimistic in imbalanced datasets, potentially reducing its discriminative power, we employed the PR curve to more accurately evaluate the model's performance, as shown in Figure 4a. The area under the PR curve reached 0.987, indicating strong capability in detecting rare anomalies. Besides, potential overfitting was examined by analyzing the free energy distributions of normal samples in both the training and test sets (see Figure S14b), which were found to be highly overlapping and to exhibit similar mean values. Figure 4b shows the free energy distributions of different sample types. The free energy of normal samples exhibited a clear Gaussian distribution, primarily concentrated in lower energy regions, indicating that the model effectively captures the typical patterns of normal data. In contrast, the free energy of abnormal samples (other digits) was more dispersed, overall higher, and exhibited a scattered distribution, reflecting the model's limited fitting ability for anomalies. This contrast in free energy provides an intuitive basis for threshold selection. We set the threshold to cover 98% of normal samples (corresponding free energy = ‐601.74), resulting in the confusion matrix shown in Figure 4c (Accuracy, Recall, Precision, and F1‐score are reported in Figure S14c). Notably, even under this high threshold, the model achieved zero missed detections (FN = 0), demonstrating its ability to effectively capture anomalous features. The ten samples with the highest free energy are shown in Figure S14d. In summary, the VM‐chip‐based RBM can efficiently capture normal patterns while distinguishing anomalies, validating its reliability and potential applicability in anomaly detection tasks.

**FIGURE 4:**
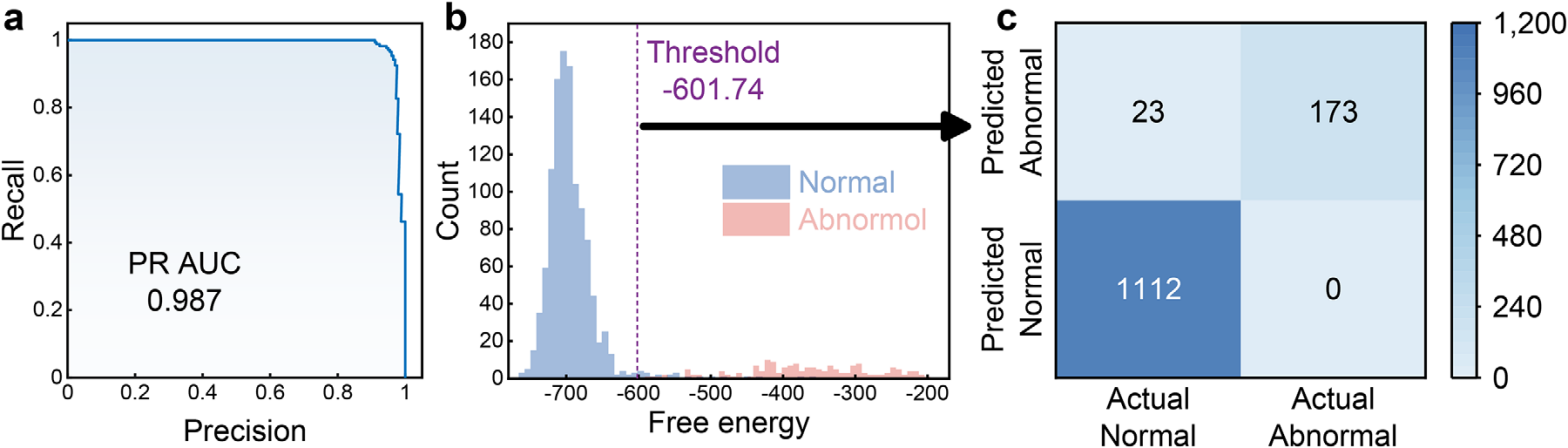
Implementation of RBM for anomaly detection based on VM‐Chip. a, PR curve obtained from anomaly detection using a DBN on the MNIST dataset, with the digit 1 treated as the normal sample. The area under the curve (AUC) is 0.987. b, Histogram of the free energy distribution for normal and abnormal samples. c, Confusion matrix based on a threshold distinguishing normal and abnormal samples, set at the 98% cumulative distribution of normal samples (free energy = −601.74).

### 2.5 VM‐chip Based Image Completion and Generation

To further evaluate the scalability of the VM‐chip‐based stochastic neural network in more complex scenarios, we additionally introduce two representative generative tasks, namely image completion and image generation. Figure 5a presents the performance of RBM under structured occlusion conditions. To construct missing regions with large area and strong spatial continuity, the upper half and right half of each input image are fixed as visible pixels, while all remaining regions are replaced with random noise. The VM‐chip then performs multiple rounds of Gibbs sampling to iteratively infer the occluded pixels and reconstruct the full image. As shown, the completed images exhibit well‐preserved stroke continuity and clearly recover the main contours of the digits. Figure 5b illustrates the model's performance under random occlusion, which emulates unpredictable corruption commonly encountered in practical applications. When the occlusion ratio is relatively low (10% to 30%), the model is able to almost perfectly recover the original digit structures, achieving high visual similarity with the original images. As the occlusion ratio increases to 50% to 70%, the original images become visually unrecognizable, the model can still extract latent cues from heavy noise and reconstruct digits with reasonable stroke patterns and global shapes. At an occlusion ratio of 90%, the extremely limited visible information inevitably introduces noticeable distortions in the completion results, yet the reconstructed digits still faintly retain their basic underlying structure. The corresponding quantitative results are shown in Figure 5c, where SSIM decreases progressively as the occlusion ratio increases. However, except for the most extreme 90% occlusion case, the model maintains reconstruction quality within an acceptable range (>0.5) across all occlusion levels. The image completion experiments demonstrate that the RBM based on VM‐chip random bit sampling can not only perform discriminative tasks but also handle more challenging generative tasks.

**FIGURE 5:**
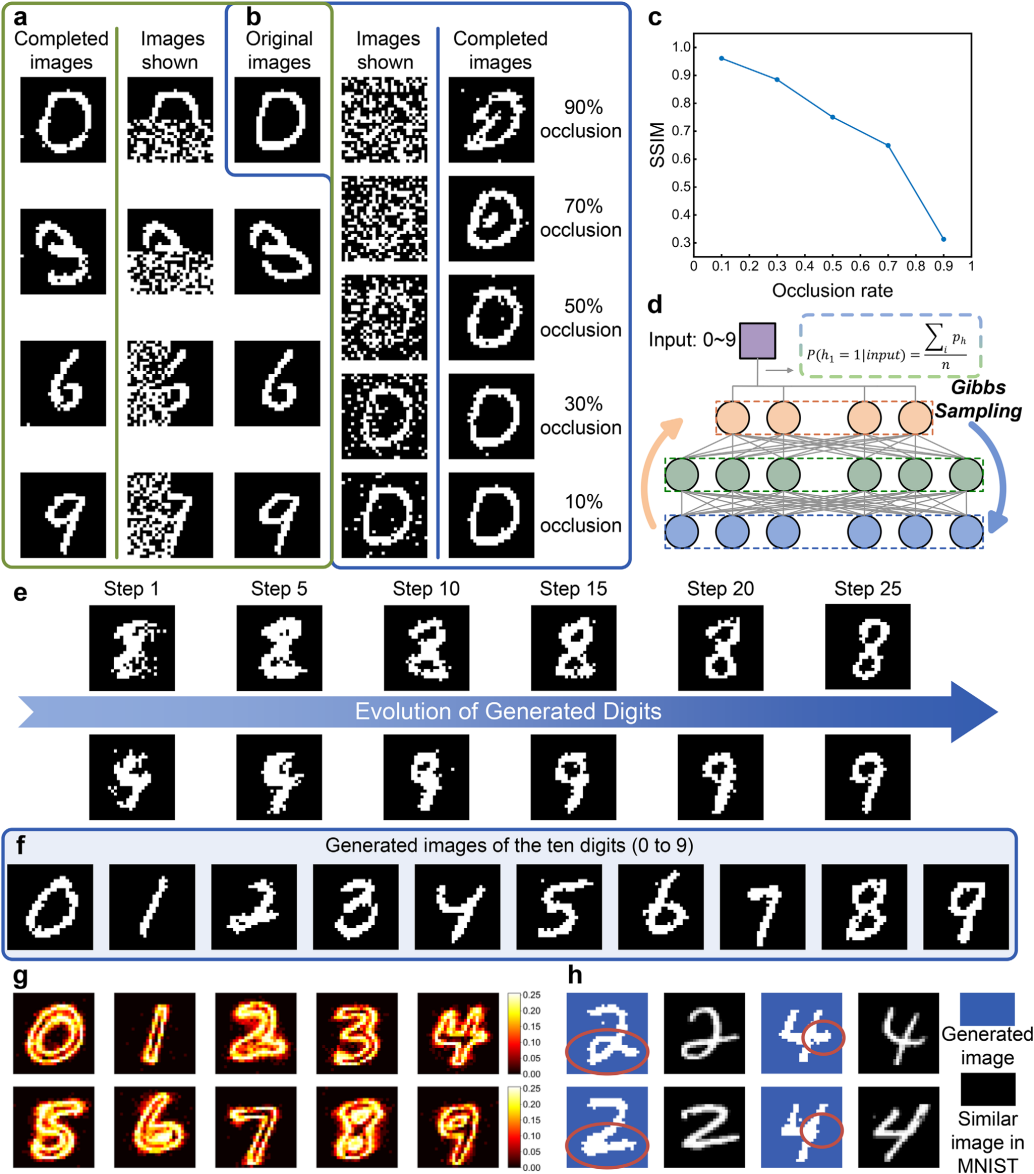
Implementation of RBM/DBN for image completion and generation based on VM‐Chip. a, Image completion examples with RBM. The left and bottom half of the images are fixed while the other half is obscured. b, Image completion examples using RBM for digit images with random occlusions of varying proportions (0.1, 0.3, 0.5, 0.7, 0.9). c, Trends of Structural Similarity Index Measure (SSIM) for image completion results at different occlusion rates. d, Schematic of the DBN model for image generation, composed of two stacked RBMs, including a visible layer and two hidden layers with 784, 512, and 256 nodes, respectively. e, Stepwise evolution of image generation by a DBN using Gibbs sampling. f, All ten digits are generated with DBN after training the network with the full MNIST dataset. g, Pixel‐wise variance heatmaps of ten digits computed from 50 consecutively generated samples. h, Diverse generated digit styles and their corresponding similar samples from the MNIST dataset. Red circles highlight the main regions of difference.

For image generation, we employed a DBN consisting of 784 visible nodes, 512 first hidden layer nodes, and 256 s hidden layer nodes, with its structural schematic shown in Figure 5d. During the training phase, the DBN not only updated parameters using the contrastive divergence algorithm but also recorded the activation probabilities of the second hidden layer units. In the generation phase, we activated the high‐probability hidden nodes corresponding to the target digit as the initial state and then gradually recovered the visible layer image through Gibbs sampling (algorithmic details in Note S7). Since the initialization state is already close to the typical activation pattern of the target class in the feature space, this approach effectively overcomes the limitation of RBM/DBN as unconditional models, which cannot directly perform targeted generation on multi‐class data. In addition, it reduces the number of sampling steps required to evolve from random initialization to a clear image, allowing clear and structurally coherent images to be generated in only 25 Gibbs sampling steps. Figure 5e illustrates the generation evolution of digits 8 and 9 (selected for display due to their structural complexity). In the initial sampling steps, the image structure is still unclear and barely recognizable. As the number of sampling steps increases, the local strokes of the digits gradually emerge and stabilize, eventually fully converging into clearly recognizable digit shapes.

The final generated results for all ten digits are shown in Figure 5f, which are free of noise, showing easily discernible shapes. The image generation experiments implemented on the DBN demonstrate that the VM‐chip‐based stochastic neural network can effectively support deep models with multi‐layer structures, validating the feasibility of using the VM‐chip for Gibbs sampling and feature extraction in multi‐layer networks. Image generation on the Fashion‐MNIST dataset can also be successfully achieved using the DBN, demonstrating that the model is capable of capturing complex image structures and generating high‐quality samples. The results are shown in Figure S15. In addition, the RBM can also generate images using the same method (see Figure S16). Compared with the DBN, RBM‐generated images contain more noise, and the pixels forming the digits are more sparsely distributed, resulting in a loosely structured and disconnected appearance. The reason lies in the fact that the RBM, as a single‐layer model, lacks high‐level structural constraints and captures only local dependencies between the visible and hidden units. To further investigate the stability of the model in consecutive image generation, we computed the pixel‐wise variance of 50 samples generated for each digit and visualized the results as variance heatmaps, as shown in Figure 5g.

The heatmaps clearly indicate that the contour regions of the digits exhibit higher variance, suggesting that the model experiences certain structural variations or minor fluctuations at these edges. In contrast, the interior regions of the digits and the background are almost completely black, indicating that the model can stably preserve the overall shape of the digits and the background information, thereby ensuring structural consistency in the generated images. Additionally, as illustrated in Figure 5h, some local regions with relatively high variance are observed in the heatmaps, which correspond to curved strokes, intersections, or endpoints of the digits, reflecting a certain degree of uncertainty in the model when handling these fine‐grained features. This local uncertainty demonstrates that while the model maintains global structural integrity, it can generate diversity in details, enhancing the richness and naturalness of the generated images. The successful completion of image generation further validates the hardware feasibility of VM‐chip‐based stochastic neural networks and provides an experimental foundation for the realization of low‐power, high‐efficiency AI hardware.

### 2.6 VM‐Chip Device‐Level Benchmarking

Table 1 benchmarks the device‐level performance of this work against existing p‐bit platforms. Considering that the energy consumption of full‐stack systems is highly dependent on the target optimization problem, algorithmic architecture, and peripheral process technology, our work adopts single‐bit intrinsic energy consumption and generation speed as the core comparison metrics. System‐level energy metrics of the VM‐chip are provided in Table S5, with a single‐bit generation energy of approximately 94 pJ/bit. To satisfy the fundamental requirement of p‐bits for continuously tunable probabilistic outputs, conventional CMOS‐based implementations inevitably rely on external bias generators, whereas the proposed approach inherently realizes this functionality at the physical‐device level, thereby eliminating the substantial area and power overhead associated with auxiliary circuitry. Compared with other spintronic mechanisms, low‐barrier sMTJs theoretically exhibit extremely low energy consumption, albeit at the expense of not only non‐volatility but also a severely limited generation speed. Benefiting from the underlying physics of VCMA, the proposed device achieves lower energy consumption and higher speed than high‐barrier MTJs dominated by STT‐ and SOT‐based switching mechanisms. Although the approach reported in Ref. [48] also exploits VCMA to modulate MTJs, its design philosophy is fundamentally different from that of the present work. In Ref. [48], 10 ns voltage pulses are employed to generate random bits with a fixed probability of approximately 50%, meaning that the MTJ serves primarily as an entropy source rather than a hardware p‐bit with continuously tunable output probabilities. Consequently, probabilistic p‐bit behavior must be reconstructed through additional ASIC‐based digital post‐processing. By contrast, the proposed VM‐chip directly realizes continuously tunable probability outputs within the VCMA‐MTJ itself, enabling the device to intrinsically perform the core function of a hardware p‐bit without requiring digital probability reconstruction, thereby achieving higher throughput, lower energy consumption, and reduced area overhead. Although an MCU is adopted in our experimental platform, it is used solely for array addressing and timing control, without participating in probability generation or p‐bit computation. These supervisory functions can be readily migrated to ASIC implementations in future system integration. Therefore, the key contribution of this work is not another demonstration of VCMA‐based random number generation, but the realization of a chip‐level hardware p‐bit with continuously tunable probabilities for stochastic neural‐network applications. For completeness, the intrinsic write energy and speed of technologically mature MRAM devices are also included as a benchmark. The evaluation results demonstrate that the VCMA‐based mechanism proposed in this work still achieves substantially lower single‐bit switching energy.

**TABLE 1:** Benchmark Comparison of Device‐Level Performance for p‐bit Platforms.

P‐bit platform	Energy (fJ)	Speed (bit/s)	Transistor count	Randomness type	Non‐volatility
This work	<30	10^9^	1T1M	True	Yes
LFSR + Bias generator [49]	234	10^8^	>1194	Pseudo	No
sMTJs [56]	2	10^3^	1T1M	True	No
STT‐MTJ [57]	100	1.25 × 10^5^	1T1M	True	Yes
SOT‐MTJ [34, 44]	>7000	2 × 10^7^	N/A	True	Yes
VCMA‐MTJ + ASIC [48]	>430	10^7^	N/A	True	Yes
STT‐MRAM [49, 58]	>120	5 × 10^7^	1T1M	N/A	Yes
SOT‐MRAM [49, 59]	>220	2 × 10^9^	1T1M	N/A	Yes

## 3 Conclusions

In summary, we demonstrate a chip‐level p‐bit array capable of running a fully functional stochastic neural network. Enabled by a tail truncation hybrid sampling method, the VM‐chip effectively compensates for device‐to‐device probability variations arising from fabrication processes, thereby improving process tolerance. This strategy may offer greater flexibility in device selection and array integration, potentially paving the way for more cost‐effective manufacturing and large‐scale production. Building upon this stable hardware foundation, we successfully implement a series of tasks ranging from image classification and anomaly detection to image completion and image generation. These tasks span a broad spectrum, covering discriminative and generative settings, low‐dimensional and high‐dimensional representations, and simple as well as increasingly complex scenarios, collectively validating the generality and robustness of VM‐chip‐based probabilistic computing. With the VM‐chip, we achieve nearly tenfold energy reduction and exponential savings in transistor resources, while providing true randomness. As probabilistic algorithms and application scenarios continue to expand, the proposed device‐level compensation strategy may facilitate the scalable integration of VM‐chip‐based probabilistic computing hardware, supporting increasingly complex neuromorphic computing tasks and future probabilistic artificial intelligence applications.

## 4 Methods

### 4.1 Fabrication and Measurement of VM‐chip

The VM‐chip was fabricated by IMEC based on its industrial‐grade, CMOS‐compatible back‐end‐of‐line (BEOL) integration platform, and monolithically integrated with 40 nm CMOS peripheral circuits. The chip layout incorporates a 32 Kb (256 × 128) VCMA‐MTJ array macro. The VCMA‐MTJs were patterned with a nominal diameter of approximately 63 nm. The switching probability versus magnetic field characteristics under varying voltage biases (Figure 1d) were obtained using a custom‐designed ultrafast probe card system. For each voltage bias condition, we performed 500 consecutive measurements to ensure statistical robustness. In the VM‐chip testing system, the Teensy 4.1 development board served as the MCU for system control and was programmed using the Arduino IDE. The VM‐chip was placed on a custom‐designed PCB. An STM32H7A3ZI development board was used solely to provide the supply voltage, triggering the on‐chip pulse generator of the VM‐chip to apply voltage pulses with predefined widths to the memory cells. For each pulse‐width condition, 1000 write‐read cycles were carried out per memory cell to extract the switching probability (Figure 1f,g).

### 4.2 Algorithmic Implementations for the Applications Presented

All implementations were carried out using Python and evaluated on the MNIST and Fashion‐MNIST datasets (60 000 training and 10 000 test samples each). For the image recognition and completion tasks, an RBM was initially unsupervisedly pre‐trained using the CD‐10 algorithm with a learning rate of 0.01. Subsequently, the recognition task extracted 256‐dimensional hidden‐layer activation probabilities to train a linear classifier (Adam optimizer, cross‐entropy loss, learning rate 0.001), while the completion task directly utilized the trained RBM for generative inference via Gibbs sampling. In the anomaly detection task, a one‐class learning setting was adopted, training exclusively on normal samples for 10 epochs at a learning rate of 0.01. For image generation, a DBN underwent greedy layer‐wise pre‐training via the CD‐1 algorithm, followed by top‐down Gibbs sampling to generate images. To ensure output consistency, a cosine similarity threshold filtering strategy was applied to the generated samples (threshold set to 0.73 for digit 5, 0.75 for digit 6, and 0.8 for all other classes).

## Author Contributions

**Jiachen Bao**: conceptualization, investigation, writing – original draft, methodology, validation, visualization, writing – review and editing, software, formal analysis, data curation. **Songsong Li**: conceptualization, methodology, software, supervision. **Ruizhi Ren**: conceptualization, methodology, software. **Zheng Zhu**: visualization, conceptualization. **Di Wu**: visualization, conceptualization. **Jiayong Wang**: visualization, conceptualization. **Minggao Zuo**: visualization. **Mingke Li**: conceptualization. **Duanzihua Cheng**: formal analysis. **Jianyu Lv**: formal analysis. **Lintong Zou**: methodology. **Chuyue Liu**: formal analysis. **Wangyang Hu**: data curation. **Yi Cao**: conceptualization, investigation, funding acquisition, methodology, validation, visualization, writing – review and editing, formal analysis, data curation, supervision, resources. **Yuesheng Li**: conceptualization, investigation, funding acquisition, methodology, validation, visualization, writing – review and editing, formal analysis, data curation, supervision, resources. **Guanghua Yu**: conceptualization, investigation, funding acquisition, methodology, validation, visualization, writing – review and editing, formal analysis, data curation, supervision, resources.

## Conflicts of Interest

The authors declare no conflicts of interest.

## Supporting information




**Supporting File**: advs78604‐sup‐0001‐SuppMat.docx.

## Data Availability

The data that support the findings of this study or additional data related to this paper are available from the authors upon request.
