# Supplementary material for: Chip‐Level Voltage Controlled Magnetic p‐Bits Array with Variation Compensation for Stochastic Neural Network Computation
Source: Adv Sci (Weinh). 2026 Nov 10:e78604. Online ahead of print. doi: 10.1002/advs.78604 (PMC13752241; doi:10.1002/advs.78604)
Supplement: Supplementary file 1 [file ADVS-9999-e78604-s001.docx]

Supplementary Information for

**Chip-level voltage controlled magnetic p-bits array with variation compensation for stochastic neural network computation**

Jiachen Bao^1^, Songsong Li^1^, Ruizhi Ren^1,4^ Zheng Zhu^2^, Di Wu^2^, Jiayong Wang^2^, Minggao Zuo^1^, Mingke Li^1^, Duanzihua Cheng^1^, Jianyu Lv^1^, Lintong Zou^1^, Chuyue Liu^1^, Wangyang Hu^1^, Yi Cao^1,3,*^, Yuesheng Li^2,*^, and Guanghua Yu^1,3,*^

*^1^School of Materials Science and Engineering, University of Science and Technology Beijing, Beijing, 100083, China*

*^2^Institute of Integrated Circuits, Shanghai University, Shanghai, 201800, China*

*^3^Key Laboratory of Advanced Materials and Devices for Post-Moore Chips, Ministry of Education, University of Science and Technology Beijing, No. 30, Xueyuan Road, Haidian district, Beijing, 100083, China*

*^4^College of Sciences, Northeastern University, Shenyang, 110819, China*

*^*^Corresponding authors:*

[*caoyi@ustb.edu.cn*](mailto:caoyi@ustb.edu.cn)*,* [*ys.albert.li@gmail.com*](mailto:ys.albert.li@gmail.com)*,* [*ghyu@mater.ustb.edu.cn*](mailto:ghyu@mater.ustb.edu.cn)

 **Supplementary Figure 1. Characterization of probabilistic switching behaviors.** **a**, Switching probability curves of VCMA-MTJ devices as a function of voltage pulse width. **b**, Corresponding sigmoidal fitting results. The red curve represents the average of the measured data.

**Supplementary Figure 2.** **Cycle-to-cycle variations of MTJ-1 and sigmoid fitting of the switching probability curves. a,** Switching probability curves measured over 100 cycles. **b,** Mean switching probabilities and standard deviations across 100 cycles, together with the sigmoid fit to the mean switching probability curve. **c,** Individual sigmoid fits to the switching probability curves of all functional devices.

**Supplementary Figure 3. Statistical characterization of random-bit sequences generated by the VM-chip.** **a,** Autocorrelation coefficients of a 10^6^ bits sequence generated at a target switching probability of P=0.5, evaluated over lags from 1 to 1000. The dashed lines indicate the approximate 95% reference bounds. **b,** Pearson cross-correlation matrix calculated from the measured random bit sequences generated by 28 individually addressed MTJ devices. The diagonal elements equal 1, while the off-diagonal elements represent pairwise cross-correlation coefficients between different devices.

**Supplementary Table 1. NIST test results for generated random bits**

| Statistical Test | P-value | Proportion | Result |
| --- | --- | --- | --- |
| Frequency | 0.035174 | 49/50 | Success |
| Block frequency | 0.657933 | 50/50 | Success |
| Cumulative sums | 0.023545/0.816537 | 49/50 | Success |
| Runs | 0.657933 | 50/50 | Success |
| Longest run | 0.534146 | 49/50 | Success |
| Rank | 0.319084 | 50/50 | Success |
| FFT | 0.455937 | 49/50 | Success |
| Non-overlapping template | - | 7335/7400 | Success |
| Overlapping template | 0.191687 | 49/50 | Success |
| Universal | 0.779188 | 50/50 | Success |
| Approximate entropy | 0.911413 | 49/50 | Success |
| Random excursions | - | 270/272 | Success |
| Random excursions variant | - | 599/612 | Success |
| Serial | 0.699313/ 0.419021 | 49/50 | Success |
| Linear complexity | 0.494392 | 50/50 | Success |

**Supplementary Figure 4. Photograph of the complete VM-chip testing system.** The Teensy 4.1 serves as the MCU, while the STM32 is used solely for voltage supply. The VM-chip is mounted on a custom-designed printed circuit board.

**Supplementary Figure 5.** **Photograph of the fabricated prototype wafer before dicing.** The wafer size is 12 inches (300 mm), fabricated using a 40 nm process node.

**Supplementary Figure 6. SEM images at various magnifications showing the circuit structure. a,** 2000. **b,** 8000. **c,** 15000. **d,** 20000.

**Supplementary Note 1. RBM and DBN model**

Restricted Boltzmann machine (RBM) is an undirected stochastic neural network based on an energy function, employing a two-layer architecture consisting of a visible layer and a hidden layer to model the probability distribution of input data. Both layers are composed of a large number of binary neurons (0 or 1), where units between layers are connected through weighted couplings while no intra-layer connections are present. The overall interactions within the network are quantified by a global energy function:

$$E(\mathbf{v},\mathbf{h})=-\sum_{i} a_{i}v_{i}-\sum_{j} b_{j}h_{j}-\sum_{i,j} v_{i}w_{ij}h_{j}$$

Here, a_i_ and b_j_ denote the biases of the visible and hidden neurons, respectively, and w_ij_ represents the weight connecting them. States with lower energy occur with higher probability, and their joint distribution follows the Boltzmann form:

$$P(\mathbf{v},\mathbf{h})=\frac{1}{Z}\exp(-E(\mathbf{v},\mathbf{h})/T)$$

Z is the partition function that normalizes the distribution so that the probabilities of all possible states sum to one. T is a temperature parameter that controls the level of stochasticity. To enable effective probabilistic computation, an RBM typically updates neuron states through Gibbs sampling, where the probability that the i-th neuron takes the value of 1 is determined by the influence of the other connected neurons and follows a sigmoid function:

$$P(v_{i}=1|\mathbf{h})=\sigma(a_{j}+\sum_{i} h_{j}w_{ij})$$

$$P(h_{j}=1|\mathbf{v})=\sigma(b_{j}+\sum_{i} v_{i}w_{ij})$$

Gibbs sampling facilitates efficient exploration of low energy regions in the state space, thereby reducing the risk of becoming trapped in local minima. Building upon this mechanism, deep belief network (DBN) constructs a deeper hierarchical architecture by stacking multiple RBM layers, where each layer is trained sequentially to learn increasingly abstract representations of the data. The joint probability distribution of a DBN can be expressed as:

$$P(\mathbf{v},\mathbf{h}^{1},\ldots,\mathbf{h}^{L})=P(\mathbf{v}\mid\mathbf{h}^{1})\left( \prod_{l=1}^{L-1} P(\mathbf{h}^{l}\mid\mathbf{h}^{l+1}) \right)P(\mathbf{h}^{L})$$

L denotes the total number of layers in the network. Each RBM within the DBN takes the activations of the hidden layer from the preceding RBM as its visible-layer input, and the conditional probabilities in every layer are computed through weighted summation followed by a sigmoid activation, consistent with the Gibbs-sampling formulation used in a single RBM. On the VM-chip, MTJs functioning as p-bits naturally support the extensive Gibbs-sampling operations required by RBMs and DBNs, while offering low power consumption and high parallelism, thereby enabling efficient deep probabilistic computation directly at the hardware level.

**Supplementary Note 2. Details of the RBM implementation**

Each image in MNIST was then flattened into a 784-dimensional vector, and data loaders were constructed with a batch size of 64 for the training set and 1,000 for the test set to facilitate mini-batch processing during model training and evaluation. The RBM consists of 784 visible nodes (with 784 visible biases) and 256 hidden nodes (with 256 hidden biases), with a weight matrix of size 784 × 256. The sampling process follows Gibbs sampling. We train the RBM using the contrastive divergence algorithm. After feeding the visible units, the hidden unit activation probabilities P are computed, and the hidden states **h** and reconstructed visible units **v** are sampled using the VM-chip. Gradients are computed according to:

$$\Delta W=\frac{1}{N}(v_{0}^{T}h_{0}-v_{k}^{T}h_{k})$$

$$\Delta a=\frac{1}{N}(v_{0}-v_{k})$$

$$\Delta b=\frac{1}{N}(h_{0}-h_{k})$$

The trained RBM is used to extract 256-dimensional hidden layer activation probabilities as features, which are then fed into a linear classifier for 10-class image classification. The classifier is trained using the cross-entropy loss function and optimized with the Adam optimizer. During each training batch, both the RBM and the classifier are updated simultaneously. The test accuracy is evaluated every 100 iterations.

**Supplementary Figure 7. Schematic of the RBM training process.** The RBM unsupervised training phase is shown in purple, and the supervised recognition phase using the RBM is shown in orange.

**Supplementary Table 2. Quantitative workload breakdown of the RBM implementation**

| Workload category | Operation | Workload | Execution |
| --- | --- | --- | --- |
| Deterministic computation | Weighted computation | 2×784×256=401,408 | Host |
|  | Gradient calculation and parameter update | 784×256 weight matrix |  |
| Stochastic computation | Hidden-state sampling | 256 states | VM-chip |
|  | Visible-state sampling | 784 states |  |
| Chip control and readout | Addressing, pulse configuration, and resistance-state readout | 1,040 sampled states | MCU |

**Supplementary Table 3. Time Breakdown of the RBM implementation**

| Component | Time (s) | Percentage |
| --- | --- | --- |
| Weighted computation | 12.68 | 29.50% |
| Gradient calculation | 1.36 | 3.16% |
| Parameter update | 0.77 | 1.79% |
| stochastic sampling | 7.80 | 18.15% |
| System/interface overhead | 20.37 | 47.40% |

**Supplementary Figure 8. Additional validation experiments using MTJ-1 generated random bits. a,** DBN classification on MNIST. The DBN is composed of three layers with 784 nodes, 512 nodes, and 256 nodes. **b,** RBM recognition on Fashion-MNIST.

**Supplementary** **Figure 9. Probability distributions during RBM sampling. a,** Reconstruction probability distribution of the visible layer. **b,** Activation probability distribution of the hidden layer.

**Supplementary Table 4. Statistical analysis of image recognition accuracy**

| **Metric** | **Software** | **MTJ** |
| --- | --- | --- |
| Mean accuracy | 0.9376 | 0.9390 |
| Standard deviation | 0.17 | 0.16 |
| Mean difference | 0.1405 | |
| *t*-statistic | 2.646 | |
| *p*-value | 0.0159 | |
| 95% confidence interval of mean difference | [0.029, 0.252] | |

**Supplementary Figure 10. Image recognition performance using individual and combined MTJ devices. a,** Image recognition accuracy obtained using each of the 28 MTJ devices individually. **b,** Image recognition accuracy across 10 independent experiments using the 28 MTJ devices jointly for stochastic computation.

**Supplementary Note 3. Integer factorization algorithm**

The computational task of factoring a targeted composite integer F is mathematically encoded by structuralizing an objective energy landscape wherein $E=(XY-F)^{2}$. To mathematically define the constituent factors, the numbers are projected via binary bit expansions as follows:

$$E=\left( \sum_{i=0}^{P} 2^{i}x_{i}\times\sum_{j=0}^{Q} 2^{j}y_{j}-F \right)^{2}$$

Here, because any non-trivial factors of an odd integer are structurally restricted to odd values, the lowest-order baseline configurations are frozen as $x_{0}=1$ and $y_{0}=1$. The dimensional boundaries of the state search are determined by the maximum bit widths P and Q.

Under this architectural mapping, a flawless mathematical factorization $XY=F$ corresponds strictly to the absolute global energy minimum $E=0$. Driven by continuous stochastic state explorations, the occupancy probability $P(x_{P},\ldots,x_{1},y_{Q},\ldots,y_{1})$ of the network configurations eventually stabilizes into a steady state governed by the Gibbs canonical ensemble:

$$P(x_{P},\ldots,x_{1},y_{Q},\ldots,y_{1})\propto exp\left( -\frac{E}{T} \right)$$

Consequently, the physical system naturally demonstrates the highest statistical residence time at the configurations possessing the lowest possible energy levels.

The underlying hardware architecture relies on p-bits to dynamically execute this invertible computing logic, with each constituent p-bit yielding a normalized binary digital output $m_{i}\in\{0,1\}$. By executing a directional derivative with respect to this output state, the localized gradient of the energy surface is directly obtained. By executing a directional derivative with respect to this output state, the localized gradient of the energy surface is directly obtained, governed by:

$$I_{i}=-\frac{\partial E}{\partial m_{i}}$$

Subsequently, the stochastic state transition of the $i$-th p-bit is executed according to the probabilistic activation rule:

$$m_{i}=\vartheta\left[ \sigma(I_{i})-r \right]$$

where $\vartheta$ denotes the Heaviside step function, $\sigma$ represents the classic sigmoidal transfer characteristics, and $r$ is an independent random variable sampled uniformly from the continuous range $[0,1]$.

In our experiment, P = 2, Q = 2, and the cost function obtained for F = 35 is：

$$\begin{aligned} E=-0.36x_{1}-0.70x_{2}-0.36y_{1}-0.70y_{2} \\ -0.59x_{l}y_{1}-1x_{l}y_{2}-1x_{2}y_{1}-1.48x_{2}y_{2} \\ +0.35x_{1}y_{1}y_{2}+1x_{2}y_{1}y_{2}+0.35x_{1}x_{2}y_{1}+1x_{1}x_{2}y_{2} \\ +0.70x_{1}x_{2}y_{1}y_{2}+0.04x_{1}x_{2}+0.04y_{1}y_{2} \end{aligned}$$

where the coefficients are rounded off to have two significant digits. The PUP required for the $i$-th p-bit equals to：

$$\sigma(I_{i})=\frac{1}{1+e^{-I_{i}}}$$

**Supplementary Note 4. Traveling Salesman Problem algorithm**

To solve the Traveling Salesman Problem (TSP), a simulated annealing algorithm was deployed and benchmarked on the standard Burma14 dataset. First, the topological configuration of the city routing sequence is represented as a state vector **S**, whose total tour length is directly mapped onto the physical Hamiltonian of the system. This mapping defines the network energy function as:

$$E(\mathbf{S})=\sum_{i=1}^{N-1} D(s_{i},s_{i+1})+D(s_{N},s_{1})$$

where $D(s_{i},s_{i+1})$ denotes the Euclidean spatial distance between consecutive cities and N = 14.

During each discrete MCMC temporal step, a candidate state **S_new_** is generated via a 2-opt neighborhood trajectory transformation. The resulting energetic gradient $\Delta E=E(\mathbf{S}_{\mathrm{new}})-E(\mathbf{S}_{\mathrm{current}})$ dynamically drives the physical flip probability of the stochastic p-bits. Guided by the Boltzmann thermal-fluctuation statistics, the idealized transition probability follows the standard Metropolis-Hastings criterion, where:

$$P_{\mathrm{ideal}}=\left\{ \begin{aligned} &1.0, &&\Delta E<0 \\ &\exp\left( -\frac{\Delta E}{T} \right), &&\Delta E\geq0 && \end{aligned} \right.$$

This segmented exponential formulation functions as the physical computing equivalent to the canonical Sigmoid activation function commonly deployed in conventional probabilistic neural networks.

This entire physical annealing lifecycle strictly obeys an exponential cooling schedule governed by an initial temperature $T_{\mathrm{initial}}=10000$, a crystallization termination temperature $T_{min}=1\times{10}^{-5}$, a cooling rate $\alpha=0.995$, and an isothermal Markov chain length of $L=100$ steps. This schedule yields an exponential decay format:

$$T_{k+1}=\alpha T_{k}$$

accumulating to approximately 4.1×10^5^ total physical sampling iterations to guarantee steady-state system convergence.

**Supplementary Figure 11.** **Experimental demonstration of integer factorization. a to b,** Distributions of factors X and Y and result statistics over 800 iterations performed by MTJ-1. **c to d,** Distributions of factors X and Y and result statistics over 800 iterations performed by MTJ-2.

**Supplementary Figure 12. Experimental demonstration of** **Traveling Salesman Problem (TSP).** **a to d,** Micro-step convergence trajectories showing the real-time stochastic exploration and history-optimal optimization paths, where **Figure** **a to d** correspond to the computer simulation, MTJ-1, MTJ-2, and MTJ-2 with the proposed tail truncation strategy, respectively. The system energy profiles are represented by Current Cost (scatter clouds) and Best Cost (solid lines). **e to h,** The corresponding final read-out topological routes.

**Supplementary Note 5. 8-Bit Digitally Controlled Pulse-Shaping Module**

The on-chip pulse-shaping module is based on a programmable RC delay circuit controlled by an 8-bit configuration word, CFG<7:0>, as illustrated in **Supplementary Figure 13a**. The circuit employs eight digitally controlled discharge branches with binary-weighted strengths. Specifically, CFG[0] controls a branch with a relative drive strength of 1×, CFG[1] controls a branch with a relative drive strength of 2×, CFG[2] controls a branch with a relative drive strength of 4×, and so on, up to CFG[7], which controls a branch with a relative drive strength of 128×. Each configuration bit determines whether its corresponding discharge branch is enabled. By combining these branches, the effective pull-down strength can be digitally adjusted across a range of configurations.

When the discharge branches are enabled, they modify the effective conductance of the delay node and thereby control its discharge rate. The time required for the node voltage to reach the switching threshold of the subsequent inverter consequently changes with the selected configuration, providing a mechanism for programmable pulse-width modulation. The 8-bit configuration provides 256 possible digital codes. The resulting pulse width depends on the effective discharge strength, the capacitance of the delay node, and the switching threshold of the inverter. Although the pulse width can be tuned over a range of discrete settings, the relationship between the configuration code and pulse width is determined by the circuit characteristics and is not necessarily perfectly linear.

To characterize the pulse-width tuning capability, transient simulations were performed using Cadence Virtuoso. As shown in **Supplementary Figure 13b**, different configuration settings produce distinct output pulse waveforms. A closer view of the simulated delay-node transitions is provided in **Supplementary Figure 13c**, illustrating the change in transition timing with the configuration setting. The simulations demonstrate a minimum pulse-width adjustment step of approximately 1 ps under the evaluated conditions. This value represents the minimum difference between programmable pulse-width settings observed in circuit simulation; it should not be interpreted as a guarantee of 1-ps absolute pulse-width accuracy at the MTJ terminals.

The generated pulses are delivered to the array through local bit-line (BL) drivers. Each 64-bit mini-array is equipped with a local driver with a drive strength approximately 64 times that of the minimum-sized driver. This sizing is intended to provide sufficient current-driving capability for the array load while limiting pulse degradation. The simulated BL waveform is shown in **Supplementary Figure13d**. At a write voltage of 1.5 V and a minimum pulse width of 0.3 ns, the simulated rise and fall times, defined between the 10% and 90% levels, are approximately 100 ps. These results characterize the simulated driver response under the specified conditions and do not constitute direct measurements of the voltage waveform at the MTJ terminals.

**Supplementary Figure 13. On-chip pulse-generation circuit and simulated pulse waveforms.** **a,** Circuit schematic of the programmable RC delay circuit, in which an 8-bit configuration word controls the binary-weighted pull-down branches to adjust the pulse width. **b,** Simulated output pulse waveforms under different configurations. **c,** Enlarged view of the simulated delay-node waveforms, illustrating the pulse-width tuning capability and the minimum adjustment step. **d,** Simulated bit-line pulse waveform, showing the rising and falling transitions under the specified driving conditions

**Supplementary Note 6. Anomaly Detection Algorithm**

In this experiment, the RBM consisted of 784 visible units, corresponding to 784 input pixels, and 128 hidden units. During training, only normal samples (digit 1) were used to train the RBM, while other digits were excluded. The contrastive divergence algorithm was employed during training, and the sampling process was implemented via the VM-chip.

Each sample was evaluated using its Gibbs free energy to determine whether it was normal or anomalous. By ranking all samples according to their free energy, those with higher free energy were considered more likely to be anomalous. A threshold was set such that samples with free energy exceeding this threshold were classified as anomalies. The underlying principle is as follows. The joint probability of the RBM can be expressed as:

$$P(v,h)=\frac{1}{Z}e^{-E(v,h)}$$

By marginalizing over the hidden layer (summing over all possible hidden states), the probability of a visible sample can be obtained as:

$$P(v)=\frac{1}{Z}\sum_{h} e^{-E(v,h)}=\frac{1}{Z}e^{-F(v)}$$

$$F(v)=-\log\sum_{h} e^{-E(v,h)}\Longrightarrow P(v)\propto e^{-F(v)}$$

The free energy is inversely related to the probability of a sample. Therefore, a higher free energy indicates a lower probability, meaning the sample deviates from the training data distribution and can be classified as anomalous. Substituting $E\left( v,h \right)$ into the equation gives:

$$\sum_{h} e^{-E(v,h)}=\sum_{h} e^{v^{T}a+h^{T}b+v^{T}Wh}=e^{v^{T}a}\sum_{h} e^{h^{T}(Wv+b)}$$

$$F(v)=-\log\left[ e^{v^{T}a}\sum_{h} e^{h^{T}(Wv+b)} \right]=-v^{T}a-\log\sum_{h} e^{h^{T}(Wv+b)}$$

Since the hidden layer units are binary (0 or 1) and independent, we have:

$$\sum_{h} e^{h^{T}(Wv+b)}=\prod_{j} \sum_{h_{j}=0}^{1} e^{h_{j}(Wv+b)_{j}}=\prod_{j} \left( 1+e^{(Wv+b)_{j}} \right)$$

Substituting this gives the formula for calculating the Gibbs free energy:

$$F(v)=-v^{T}a-\log\prod_{j} \left( 1+e^{(Wv+b)_{j}} \right)=-\sum_{i} v_{i}a_{i}-\sum_{j} \log(1+e^{(Wv+b)_{j}})$$

The term $-\sum_{i} v_{i}a_{i}$ corresponds to the visible layer component. It depends only on the visible layer inputs and biases and measures how well the input sample matches the visible layer biases. The term $-\sum_{j} \log(1+e^{(Wv+b)_{j}})$ corresponds to the hidden layer component and measures the ability of the hidden layer to explain the input sample.

In this application, the nodes of the stochastic neural network are implemented using MTJs. We evaluated the implementation using each of the 28 MTJ devices individually and further evaluated the network using all 28 devices jointly. In both cases, each device was operated with its corresponding tail-truncation strategy.

**Supplementary Figure 14. Implementation of RBM for anomaly detection based on VM-Chip a,** ROC curve obtained from anomaly detection using RBM on the MNIST dataset, with the digit 1 treated as the normal sample and all other digits treated as anomalous samples. The dashed line represents the diagonal of a random classifier, and the area under the curve (AUC) is 0.998. **b,** The free energy distributions were used to verify potential overfitting. **c,** The table of Precision, Recall, F1-score and Accuracy. **d,** Top 10 ranked samples.

**Supplementary Note 7. Image Generation Algorithm**

In this work, we employ a Deep Boltzmann Machine (DBN) for digit image generation. The DBN consists of two stacked RBMs: the first RBM (RBM1) has a 784-512 structure, and the second RBM (RBM2) has a 512-256 structure, forming a three-layer network corresponding to the visible layer v, first hidden layer h_1_, and second hidden layer h_2_. The MNIST dataset is first binarized and then fed into the visible layer. The training procedure is as follows: RBM1 is trained first, and its hidden layer activations are then used as the visible layer input for RBM2; details of the training procedure are provided in Supplementary Note 1. Each RBM is trained for 5 epochs. After training, we compute the activation probabilities of each node in **h_2_** and calculate their mean values, as described by the following formula:

$$P(h_{1}=1|input)=\frac{\sum_{i} p_{h}}{n}$$

The **h_2_** state is first sampled based on the mean vector of the target digit, and the visible layer image is then iteratively reconstructed via Gibbs sampling. Due to the inherent stochasticity of the generative process, a self-consistency check is introduced to ensure reliability: only images whose top-layer hidden representations exhibit a cosine similarity above a predefined threshold with the target digit mean and are correctly classified are retained. The cosine similarity is computed using the following formula:

$$s_{c}=\cos(\mathbf{h}^{(gen)},\boldsymbol{\mu}_{c})=\frac{\mathbf{h}^{(gen)}\cdot\boldsymbol{\mu}_{c}}{\parallel\mathbf{h}^{(gen)}\parallel_{2}\left\| \boldsymbol{\mu}_{c} \right\|_{2}}=\frac{\sum_{i=1}^{d} h_{i}^{(gen)}\mu_{c,i}}{\sqrt{\sum_{i=1}^{d} (h_{i}^{(gen)})^{2}}\sqrt{\sum_{i=1}^{d} (\mu_{c,i})^{2}}}$$

$\mathbf{h}^{(gen)}$is the top-layer (**h_2_**) hidden vector of the generated image, $\boldsymbol{\mu}_{c}$is the top-layer mean vector of the target digit c and d=256 is the number of h_2_ nodes. The value of $s_{c}$ ranges from -1 to 1, with values closer to 1 indicating that the generated vector is more similar to the mean vector of the target class. The selection criterion for the self-consistency check is given by:

$$\mathrm{accept}(\mathbf{h}^{(gen)})=\left\{ \begin{aligned} &\mathrm{True}, &&\mathrm{if}\hat{c}=c \mathrm{and}s_{c}\geq\tau_{c} \\ &\mathrm{False}, &&\mathrm{otherwise} && && && \end{aligned} \right.$$

Let $\hat{c}$ denote the predicted class of the generated image, and $\tau_{c}$ represent the predefined threshold.

In this application, the nodes of the stochastic neural network are implemented using MTJs. We evaluated the implementation using each of the 28 MTJ devices individually and further evaluated the network using all 28 devices jointly. In both cases, each device was operated with its corresponding tail-truncation strategy.

**Supplementary Figure 15. Image generation performed on the Fashion-MNIST dataset with DBN.** Since the Fashion-MNIST dataset becomes difficult to recognize after binarization, the visible layer takes the original image vectors as input, while its output represents the activation probabilities of the visible nodes (grayscale values). During training, parameter updates are still performed using binarized samples.

**Supplementary Figure 16. Image generation performed on the MNIST dataset with RBM.** Compared with the DBN, the pixels forming the digits in RBM generated images are more sparsely distributed, resulting in a fragmented overall structure.

**Supplementary Table 5. System-level energy metrics of the VM-chip**

| Power | 37.5mW |
| --- | --- |
| Clock Frequency^†^ | 50 MHz |
| Throughput^†^ | 400 MHz |
| Full-Chip Energy Consumption^‡^ | ~94 pJ/bit |
| Read/Write Circuit Energy^‡^ | <0.8 pJ/bit |

^†^The chip architecture employs an 8-bit parallel design, enabling the concurrent generation of 8 random bits within a single clock cycle. At a system clock frequency of 50 MHz, this corresponds to a theoretical maximum output throughput of 400 Mbit/s under fixed-probability operation. When independently programmable p-bit sampling is required, additional time may be needed to configure the target probabilities, which can reduce the effective throughput if configuration and random-bit generation cannot proceed in parallel. If probability configuration can be performed concurrently with ongoing data generation, the output throughput may be maintained during reconfiguration.

^‡^Energy dissipation analysis indicates that the intrinsic core unit (the spin device and its local microelectronics) consumes less than 1% of the total energy. The vast majority of the energy originates from peripheral control circuits, I/O pads, and auxiliary testing interfaces utilized for characterization.
